# Data for improvement and clinical excellence: protocol for an audit with feedback intervention in long-term care

**DOI:** 10.1186/1748-5908-5-74

**Published:** 2010-10-13

**Authors:** Anne E Sales, Corinne Schalm

**Affiliations:** 1 Faculty of Nursing, University of Alberta, 6-10 Terrace Building, Edmonton, AB, T6G 2T4, Canada; 2 Shepherd's Care Foundation, 6620-28 Avenue, Edmonton, Alberta, Canada

## Abstract

**Background:**

There is considerable evidence about the effectiveness of audit coupled with feedback, although few audit with feedback interventions have been conducted in long-term care (LTC) settings to date. In general, the effects have been found to be modest at best, although in settings where there has been little history of audit and feedback, the effects may be greater, at least initially. The primary purpose of the Data for Improvement and Clinical Excellence (DICE) Long-Term Care project is to assess the effects of an audit with feedback intervention delivered monthly over 13 months in four LTC facilities. The research questions we addressed are:

1. What effects do feedback reports have on processes and outcomes over time?

2. How do different provider groups in LTC and home care respond to feedback reports based on data targeted at improving quality of care?

**Methods/design:**

The research team conducting this study comprises researchers and decision makers in continuing care in the province of Alberta, Canada. The intervention consists of monthly feedback reports in nine LTC units in four facilities in Edmonton, Alberta. Data for the feedback reports comes from the Resident Assessment Instrument Minimum Data Set (RAI) version 2.0, a standardized instrument mandated for use in LTC facilities throughout Alberta. Feedback reports consist of one page, front and back, presenting both graphic and textual information. Reports are delivered to all staff working in the four LTC facilities. The primary evaluation uses a controlled interrupted time series design both adjusted and unadjusted for covariates. The concurrent process evaluation uses observation and self-report to assess uptake of the feedback reports. Following the project phase described in this protocol, a similar intervention will be conducted in home care settings in Alberta. Depending on project findings, if they are judged useful by decision makers participating in this research team, we plan dissemination and spread of the feedback report approach throughout Alberta.

## Background

The evidence for specific interventions to implement evidence-based practices in various healthcare settings is mixed at best [1-6]. Many interventions have been rigorously tested across multiple settings and conditions, and some evidence exists for their use in implementing evidence-based practice [7-9]. One of these is the use of audits combined with feedback reports.

Audit of performance, including both process and outcome measures, is an essential but probably insufficient condition for any quality improvement effort. Without audit of key indicators, it is not possible to assess the quality of care being provided. Audit requires access to data regarding processes and outcomes of care, and may require additional data elements depending on the sophistication of the audit system, the audit targets, and the indicators being monitored. As the evidence-based care movement has developed over the last several years in Canada and other developed countries, audit has played a major role in providing information about adoption of evidence-based practices in many settings and contexts.

When coupled with some form of feedback mechanism in which data are fed back to providers, audit becomes the backbone of one of the most commonly applied and widely tested initial methods of achieving quality improvement or attempting to facilitate the adoption of evidence-based practices. There is considerable evidence about the effectiveness of audit coupled with feedback, although few audit with feedback interventions have been conducted in long-term care (LTC) settings to date. In general, the effects are modest at best, although in settings where there has been little history of audit and feedback, the effects may be greater, at least initially [7,8]. The probable mechanism by which audit with feedback has its effect is in providing people with information about their own performance [3,10-13]. The results, particularly with people who have not received data-based feedback on their performance in the past, may be to provide a mild incentive to change behavior [12]. Coupling feedback with benchmarks, or information to allow providers to assess themselves in comparison to other providers or groups, may improve the effectiveness of audit with feedback. There is not much evidence about how audit with feedback works in the context of complex healthcare organizations.

There is a wide range of possible outcomes that may be affected by interventions to implement evidence-based practices. These include patient or resident outcomes (improved care, such as improved pain management, improved falls risk assessment and intervention, or improvements in managing problem behavior exhibited in dementia), provider outcomes (improved job satisfaction, improved research utilization), and system outcomes (lower staff turn-over, lower costs of care). In addition, process outcomes may be relevant in assessing whether or not interventions are fully implemented. Process outcomes include measures of uptake of feedback reports, numbers of staff attending education sessions, and intent to change behavior [14,15]. This latter measure, intent to change behavior, may mediate observable behavior change. Measuring intent to change behavior among providers who are the target of interventions to implement evidence-based practices offers an opportunity to assess whether this important initial step was met or not. Similarly, self-reported research utilization may be a mediator for observable change in practice [16-22]. Measuring self-reported research utilization also offers an opportunity to assess uptake of research evidence.

### Primary purpose and objectives

The primary purpose of the Data for Improvement and Clinical Excellence (DICE) Long-Term Care project is to assess the effects of an audit with feedback intervention delivered monthly over 13 months in four LTC facilities, using data from the Resident Assessment Instrument (RAI).

We address these research questions:

1. What effects do RAI feedback reports have on processes and outcomes over time?

2. How do different provider groups in LTC and home care respond to feedback reports based on RAI data targeted at improving quality of care?

## Methods/design

The overall intervention evaluation uses a controlled interrupted time series design with monthly feedback reports in nine LTC units in four facilities. Surveys to assess uptake of the audit with feedback intervention are conducted one week after feedback report distribution. The purpose of this survey is not to assess change in behavior, but intent to change, as well as to assess staff response to the feedback reports.

The process evaluation, conducted concurrently with the prospectively collected survey data, uses observation and self-report to assess uptake of the feedback reports. We define uptake as reading the feedback reports, discussing with colleagues and managers, and reporting some degree of intention to change behavior based on the reports.

This project has received ethics approval from the Health Research Ethics Board, Committee B, at the University of Alberta, and operational approval from the two LTC organizations participating in the study.

### Project team

The project team comprises both researchers and decision makers; team member details are provided in Appendix A (additional file 1). The specific program funding for this project requires active collaboration between researchers and decision makers (http://www.chsrf.ca/funding_opportunities/reiss/index_e.php), and the team works on a linkage and exchange, integrated knowledge translation model. Our team existed before this project was conceived, and most members had considerable experience working together in a project called the Knowledge Brokering Group (KBG), a network of Alberta healthcare decision makers and researchers that focused on data-driven approaches to improving quality of care in continuing care settings. KBG was funded for three years from 2004 through 2007, and sponsored several researcher-decision maker collaborative projects, as well as a newsletter, breakfast series, and other events such as workshops and conferences. Much of its work focused on the implementation and application of RAI data to continuing care settings in Alberta.

### Settings and sample

The settings are nine LTC nursing units in four facilities or nursing homes (NHs) in Edmonton, Alberta, Canada. The facilities have all implemented the Resident Assessment Instrument Minimum Data Set (RAI) version 2.0 (http://www.interrai.org).

## The intervention

### Procedures for feedback report generation and distribution

We include facility administrators, nurse managers, and front-line direct-care staff, including registered nurses, licensed practical nurses, nurse aides (also called healthcare aides), physical therapists, recreational therapists, occupational therapists, pharmacists, social workers, and other allied health providers. We use the TREC survey [23] to assess context in the facilities and units. This survey was administered at baseline, prior to beginning report distribution, and again at the end of the 13-month intervention period. Unlike previous studies, the reports are focused on unit-based staff, rather than the whole facility [24]. The goal of the feedback report distribution is to ensure that front-line staff receive the reports directly.

The feedback reports were developed during a pilot study conducted in two NHs in the Edmonton area in late 2007 and early 2008. We use data from the RAI 2.0 as the source data for the feedback reports as well as to measure resident-level outcomes. The RAI 2.0 covers a wide range of process and outcome data at the individual resident level, and assessments are generally updated quarterly for each resident unless there is a new admission, or a major change in a resident's demographics or in functional or cognitive status. We report on measures of pain frequency and intensity, occurrence of falls, and depression prevalence, all aggregated to the unit level. These three areas are among the top eight domains identified as important by LTC staff through the pilot project, and were agreed upon by senior leadership in both participating organizations. Data are extracted from each facility at the resident level, without personal identifiers except for the unit in which each resident lives. We use only data from assessments completed in the month being reported to ensure that reports cover current status for residents. Reports provide data from four months previously, the most current data we could process into reports, given the time it takes for assessments to be completed and processed through the vendor software. Data are obtained directly from the vendor by staff at the participating organizations, de-identified, and made available to our research team.

Reports are primarily graphic with minimal text bullets, contained on one sheet of paper front and back, printed in color. A cover sheet is always included that provides details about the data and the comparison units. An example is provided as Appendix B (additional file 2). The first monthly report provided single point in time comparisons for each unit compared to the combined other eight units. After the first monthly report, we began showing data as monthly points with a trend line joining the points. We used this approach from months 2 to 11, after which we switched to showing quarterly time points for months 12 and 13. We changed approaches for two reasons: first, we were interested in evaluating whether the different graphical presentations affected the proportion of staff of different types who reported understanding the reports; and second, we changed to quarterly time points to make the intervention sustainable by the organizations participating in the intervention. The software used to collect RAI 2.0 assessments in these facilities permits time aggregation quarterly, but not monthly without specific programming to process the data. A separate but related concern on the part of the research team was that estimates were not always stable each month, as relatively few new assessments were conducted each month.

Reports are hand delivered by project staff in each of the nine nursing units during a consistent week in each month during the 13 months of the intervention period. Each report is specific to the nursing unit, and all direct care providers of all disciplines and groups, and managers in each unit, receive the unit-specific reports. Facility administrators receive reports for each of their units prior to report distribution on the units. Hand delivery is accomplished by a research assistant visiting the unit, and handing out feedback reports directly to providers who are working at the time of delivery. Reports are put into mailboxes or left in breakrooms for providers not working during delivery periods. Two research assistants visit each unit at the same time to deliver reports. One research assistant observes the behavior of staff as they receive reports, and maintains counts of specific behaviors (observation form provided in Appendix C (additional file 3)), for example, whether the staff member reads the report immediately, or puts it into his/her pocket instead of reading immediately. We use counts of staff reading or looking at the feedback reports, as well as staff self-report on the surveys administered after feedback report delivery to estimate uptake of the reports.

In addition to the intervention delivered to the nine LTC units in the four participating LTC facilities, we will also request data from the same period for four additional facilities matched, as closely as possible, to the two organizations participating in the study. These will provide comparison data to check for secular trend over the intervention and follow up periods.

### Process evaluation

We conduct surveys of all staff in the four facilities to assess response to feedback reports. Surveys are conducted one week after feedback reports are distributed in each facility. Research assistants visit each unit within each facility, and offer all staff the opportunity to complete the post-feedback survey. Although throughout the intervention period we have generally conducted monthly post-feedback report surveys, we elected to skip months in the summer and over the holiday season to prevent survey fatigue, and avoid increasing pressure on staff during low staffing periods. As a result, while we have 13 monthly report distributions in the intervention period, we will have nine post-feedback report surveys. Staff take time during their shifts to come to a central location to complete the survey using pen and paper. Surveys are anonymous, identifying only nursing unit and facility where the staff member works, and type of provider.

Surveys include questions to assess whether staff received reports, whether they read them, whether they used them in their daily work to attempt to improve care to individual residents; if so, what kinds of actions were taken, and whether formal efforts at quality improvement were initiated, as well as less formal efforts. These questions all address issues of uptake of the feedback reports. We also ask about barriers encountered in the receipt, reading, and use of reports, as well as facilitative features of context and activities within the NHs. The last section of the survey is intended only for staff who provide direct care to residents, and focuses on the intent to change behavior, with the focal behavior being intent to assess pain among the residents the staff member cares for. These questions were constructed using a manual that describes how to construct a survey to measure key constructs from the Theory of Planned Behavior [25,26]. The survey instrument is included as Appendix D (additional file 4).

### Process outcomes

Our objective in conducting the process evaluation is to assess uptake of feedback reports and staff self-reported intent to change behavior. One of the most commonly observed reasons for failure of a knowledge translation or implementation intervention is lack of uptake of the intervention [27-31]. Without a contemporaneous process evaluation, it is usually infeasible to assess the degree of uptake of the intervention. We have discussed the rationale for measuring intent to change behavior earlier. Including intent to change behavior as an intermediate process outcome will assist in assessing whether, despite reading and understanding the feedback reports, staff do not perceive a need to change behavior.

## Analysis

We will use both quantitative and qualitative approaches to analyze data from this study.

### Quantitative analysis

We will analyze RAI 2.0 data from all nine units in four facilities to assess resident outcomes. Data in the intervention facilities are extracted monthly during the intervention period to facilitate feedback report generation. Data will be extracted in the control facilities at the end of the post-intervention surveillance period, and will be analyzed after this period. Our primary analysis, using time series with and without adjustment for covariates, including unit level context, will allow us to assess change related to delivery of a feedback report over time. We will assess outcomes included in the feedback reports (pain, depression, and falls) and other outcomes not included in the reports (*e.g*., pressure ulcers, incontinence, and social engagement).

We will measure each intervention episode (delivery of reports), and chart these graphically with the time series. This will provide a graphic depiction of changes in outcomes over time and follows the approach used in a previous study [32]. We will analyze the data using interrupted time series to assess the impact of feedback reports. We will construct aggregate measures at the nursing unit level, including proportion of residents with uncontrolled pain, recent falls, and symptoms of depression, at monthly intervals, beginning as far back as possible using available data. We anticipate having at least 12 months of data prior to the intervention period, and at least 12 months after the intervention ends, together with 13 months within the intervention period. The primary predictor variable in these analyses will be the dose of intervention, measured as the proportion of staff who are observed or who self-report reading the feedback reports, measured through the formative evaluation at the unit or facility level. All multivariate regression analyses will use cluster correction to adjust for the effect of unit and facility. With nine units in four facilities, we have too few units to use full hierarchical modeling. However, we will estimate the intra-cluster correlation coefficients for key outcomes and variables, which will assist future researchers in estimating sample size for similar unit-based interventions in LTC.

### Analysis of qualitative process evaluation data

We will code themes, specific barriers, and facilitators, and use the data from post-feedback interviews to assess degree of penetration of reports, problems with penetration, degree to which reports were used by which types of staff, actions taken in response to reports, and other information from the interview data. We will count the number of times themes recur as one quantitative measure from the qualitative data, and merge counts, at the unit level, with outcomes data from the RAI 2.0, to assess the impact of uptake of the audit with feedback intervention on resident-level outcomes using multi-level regression modeling to adjust for clustering by resident.

### Timeline

The audit with feedback intervention in the four NH facilities began in January 2009 and will continue until February 2010. The second phase of the overall DICE project, implementing a feedback intervention in home care settings using the RAI instrument designed to assess clients receiving long-term home care services (RAI-HC) will begin in fall 2010. Following a yearlong intervention with quarterly report distribution to several home care offices, the DICE project will enter its final year, focusing on dissemination and spread of the intervention throughout the province of Alberta.

### Dissemination and spread

As noted in the timeline, we will spend the final year of the program implementing the tools developed through the research conducted in the first three years. We will develop toolkits and training materials. Decision makers on the team will guide us in recruiting participation throughout the province for the implementation effort. A number of health authority representatives and LTC organizations approached DICE decision-maker research team members about interest in and willingness to continue engagement in a network focused on use of RAI data. This network was funded through a separate project by the Canadian Institutes for Health Research (CIHR), *Putting RAI to work: Network of RAI data users and researchers*, funded from 2008 to 2010 (http://www.rairesun.ca/).

One of the factors affecting Alberta's healthcare system at the time of this project was a large-scale reorganization of the healthcare system that began in April 2008, and is still being formalized in mid-2010. The nine regional health authorities were disbanded and centralized into a single provincial health authority (Alberta Health Services), which now consists of five geographic zones (http://www.albertahealthservices.ca/204.asp). The organizational structure of Alberta Health Services consists of a matrix with province-wide strategic management and planning, and ongoing operations managed through the geographic zones (http://www.albertahealthservices.ca/files/org-orgchart.pdf).

We believe that we will have a ready group of willing zones and organizations to participate in dissemination and spread activities. We will approach senior leadership in each zone and solicit their participation. If the zone is willing to participate, we will approach the administrators of the LTC facilities as well as the local home care services leadership to request their participation. Participation by facilities and home care services will be voluntary. We will offer the RAI coordinators in each facility and home care office the tools and training in how to create feedback reports, as well as guidance in delivering reports, and lessons learned from the research in Edmonton. We will continue to offer technical assistance through the next six to eight months as they implement a program of feedback reports.

We will evaluate the implementation effort through two approaches. First, we will conduct a one-time survey in each participating facility, with all willing staff, to assess response to the feedback reports. Second, we will request RAI 2.0 and RAI-HC data for the participating local health authorities to assess changes from the year prior to the implementation of the feedback reports to six months after the training, to enable us to complete the analyses during the funding period. If we are successful in securing additional funding for further work, we will extend the monitoring period. Key researchers will take a lead role in delivering this implementation plan, and will participate in site visits to each of the participating facilities in the regions with the research assistant. The site visits will be coordinated with distribution of feedback reports, which will be the responsibility of the RAI coordinators in the zones and facilities. During these visits, the researchers and RA will administer post-feedback surveys to assess feedback report distribution, uptake, perceived usefulness, and intent to change behavior. We will monitor actual outcomes using RAI data from the provincial data repository, due to become available in 2011.

A provincial project now underway will help pave the way for these dissemination activities. Six of the DICE project team members are involved in the committee overseeing the LTC Quality Improvement Project funded by Alberta Health and Wellness to provide support to LTC facilities in using RAI data for quality improvement. In that project, facilities have been provided with access to quality consultants to learn how to use their data and to implement quality improvement processes. This support will lay the groundwork for facilities to see the value of using these data, which will create interest in using feedback reports.

## Deliverables

1. A robust, replicable process for identifying quality improvement priorities across provider groups that will reliably develop actionable feedback reports;

2. A toolkit, including a manual and programming guides, to create actionable quality improvement feedback reports from RAI data;

3. A functional web site to deliver tools for assessing priorities, creating feedback reports, and delivering a feedback intervention based on data from RAI-MDS 2.0 and RAI-HC tools;

4. A cadre of decision makers and researchers who are well-versed in developing and using these tools within diverse continuing care settings.

We will use findings from this study to identify best practices and implement process improvements in the use of RAI clinical data. We believe our work will be an important contribution to the care delivery community. We expect the results of this study to be widely applicable and useful to managers in many jurisdictions, well beyond Alberta. In addition to providing important guidance about use of feedback reports in LTC settings, our highly structured approach may provide some guidance to researchers in implementation science in terms of organizing and planning audit with feedback interventions.

## Competing interests

The authors declare that they have no competing interests.

## Authors' contributions

AES conceived of the study, drafted, and revised it, and is responsible for its conduct. CS conceived of the study, reviewed, and contributed to drafts, and shares responsibility for its conduct. All authors read and approved the final manuscript.

## Supplementary Material

Additional file 1 **Team Description**. This file contains a brief description of the members of the research team and their role in the project. Click here for file

Additional file 2 **Example of Feedback Report**. This file provides an example of the type of feedback report distributed to staff as part of the intervention in this project. Click here for file

Additional file 3 **Observational checklist**. This file contains the checklist used to assess staff behavioural response to the feedback report at the time of distribution. Click here for file

Additional file 4 **Post-feedback Survey**. This file contains an example of the survey administered to staff in the long term care facilities a week after report distribution. Click here for file
