# Supplementary material for: Data for improvement and clinical excellence: protocol for an audit with feedback intervention in long-term care
Source: Implement Sci. 2010 Oct 13;5:74. doi: 10.1186/1748-5908-5-74 (PMC2964554; doi:10.1186/1748-5908-5-74)
Supplement: Additional file 1 — Team Description. This file contains a brief description of the members of the research team and their role in the project. [file 1748-5908-5-74-S1.PDF]

Appendix A: Project team member descriptions at the time of proposal funding[1]

Anne Sales, RN, PhD, is Research Lead. She is Associate Professor in the Faculty of Nursing and Canada Research Chair in Interdisciplinary Healthcare Teams at the University of Alberta. The proposed project is closely related to her other current projects, and relates closely to her Canada Research Chair. Her research program includes a strong emphasis on highly applied research in quality improvement using existing data. She will take the lead in designing and conducting the research project, as well as in translating findings both in the practice and policy communities.

Corinne Schalm, MSG, MPA, is Decision-Maker Lead. She is the Vice President of the Shepherd's Care Foundation, Edmonton, Alberta. She is a Certified Health Executive with over 20 years experience in health care planning and administration. She has been a frequent participant on research teams; she was a key participant in the CHSRF-funded Knowledge Brokering Group (KBG) in the Edmonton area. She will participate in the design and conduct of the research project, and take the lead in disseminating findings to the decision-maker and policy communities both in Alberta and nationally.

Marian Anderson, RN, GNC is the Director of Care and Site Lead at Shepherd's Care Millwoods Care Centre. She is a decision-maker, and will assist in conducting the project as well as disseminating to nursing directors in continuing care organizations; she will also contribute to capacity-building by helping to develop training programs for managers and frontline providers.

Kari Elliott, MS is Director, Quality Management and Planning, in the Home Care Division in Capital Health, Edmonton. She is a decision-maker and will participate in conducting the project

[1] Several team members have changed positions since the proposal was first submitted. We provide the information current at the time of proposal submission.

in home care settings as well as in knowledge translation activities with home care professionals; with Ms. Anderson, she will also assist in capacity-building through training.

Carole Estabrooks, PhD, is Professor in the Faculty of Nursing and Canada Research Chair in Knowledge Translation at the University of Alberta. She is a researcher, and will participate in conducting the research and in disseminating through academic channels as well as through the practice based research network she is developing in continuing care. Her expertise in knowledge transfer and exchange provides a significant asset to the team.

Vivien Lai, MA, is the Senior Policy Advisor in the Strategic Directions Division of Alberta Health and Wellness. She is a decision-maker at a senior policy level, and will provide advice and consultation throughout the project and its development, and participate in knowledge transfer and exchange in the practice and policy communities.

Lili Liu, PhD, is Associate Professor and Acting Chair of the Department of Occupational Therapy, Faculty of Rehabilitation Medicine at the University of Alberta. She is a researcher, and will participate in conducting the research and in dissemination, particularly through her strong connection to the rehabilitation health care community.

Suzanne Maisey, MA, is Director of Quality Improvement Projects, Shepherd's Care Foundation, Edmonton, Alberta. She is a decision-maker, and will participate in designing and conducting the project, as well as in knowledge transfer and exchange in the practice community; she was also an active participant in the CHSRF-sponsored KBG.

Lynne Mansell, MHSA, is Director of Planning in the Community Care, Rehabilitation and Geriatrics Division of Capital Health. She is a decision-maker partner, and will participate in conducting the project in home care settings, and will also disseminate findings through the practice and policy communities.

Colleen Maxwell, PhD, is Associate Professor in the Departments of Community Health Sciences (Centre for Health & Policy Studies) and Medicine (Division of Geriatric Medicine), University of Calgary. She is a researcher, and will participate in disseminating findings both through traditional academic channels and through practice and policy channels; her contacts in other regions in Alberta will be invaluable in knowledge transfer and exchange; she is well-known throughout the province because of an existing project in LTC and assisted living.

Iris Neumann, BSc MSc, is Chief Executive Officer of CapitalCare. She is a decision-maker and will participate in knowledge transfer and exchange in the decision-maker and policy communities.

Sharon Warren, PhD, is Professor and Director of Rehabilitation Research in the Faculty of Rehabilitation Medicine at the University of Alberta. She is a researcher and will participate in designing and conducting the study, and in disseminating findings through the research community, particularly in rehabilitation health care.
