# Supplementary material for: Data for improvement and clinical excellence: protocol for an audit with feedback intervention in long-term care
Source: Implement Sci. 2010 Oct 13;5:74. doi: 10.1186/1748-5908-5-74 (PMC2964554; doi:10.1186/1748-5908-5-74)
Supplement: Additional file 2 — Example of Feedback Report. This file provides an example of the type of feedback report distributed to staff as part of the intervention in this project. [file 1748-5908-5-74-S2.PDF]

Data for Improvement and Clinical Excellence (DICE)

- We have developed feedback reports for staff that come from the Resident Assessment Instrument (RAI)-Minimum Data Set (MDS) information to help staff in long term care facilities improve the quality of care for residents.

- Data are being collected from 4 long term care facilities in Edmonton. The MDS data that you see in this report compares your unit to the 8 other units included in this project.

- We will hand out the feedback reports every month for 12 months beginning in January 2009. The feedback report you have now is the 8th report distributed in August 2009. The MDS data you see in this report comes from July 2008 to April 2009.

- The graphs are in 2 colors – bright green represents all other units from the 4 facilities participating in the study and the other color is for your unit. Unlike the 1st feedback report where you saw bar charts, the 2nd feedback report and the succeeding reports display a line representing time measured in months.

- Each point in the line shows a percentage, which is one form of a proportion. It is the number of residents with the health condition divided by the total number of residents who have had a new assessment for a particular month. A proportion is calculated each month for your unit and one for the 8 other units.

- The numbers at the right side show the total number of residents included each month; residents have a MDS assessment done every three months, so each month about one third of the residents on your unit have a new assessment done.

- We would like you to read the feedback report, and if you have time and are willing, please participate in a survey when we return to your facility next week. The survey takes about 10 minutes to complete.

- If you have any questions about the survey, you can contact:
  - Anne Sales (492-8432), the principal investigator of this study or
  - Gloria Gao (492-2956), the project coordinator

DICE Project
Feedback Report, August 2009

➢ PAIN

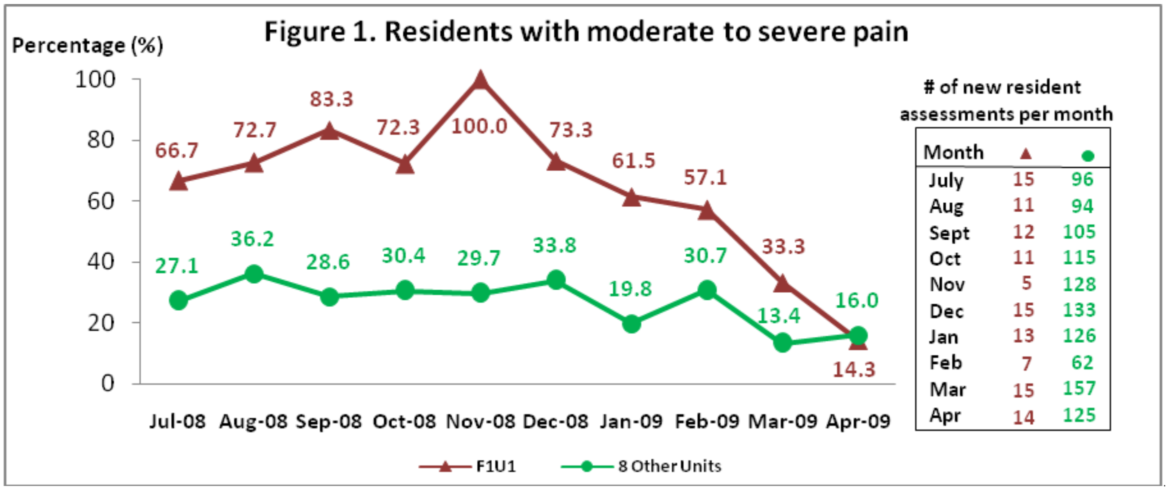

Figure 1. Residents with moderate to severe pain

- The information in Figure 1 is from the Health Conditions section (Section J) of the RAI-MDS 2.0, items J2a and J2b
- The pain scale uses these two items to measure how often and how much pain is experienced by the residents
- In April, there was a large decrease in the proportion of residents in F1U1 with moderate to severe pain; it was about the same as the other 8 units

➢ DEPRESSION

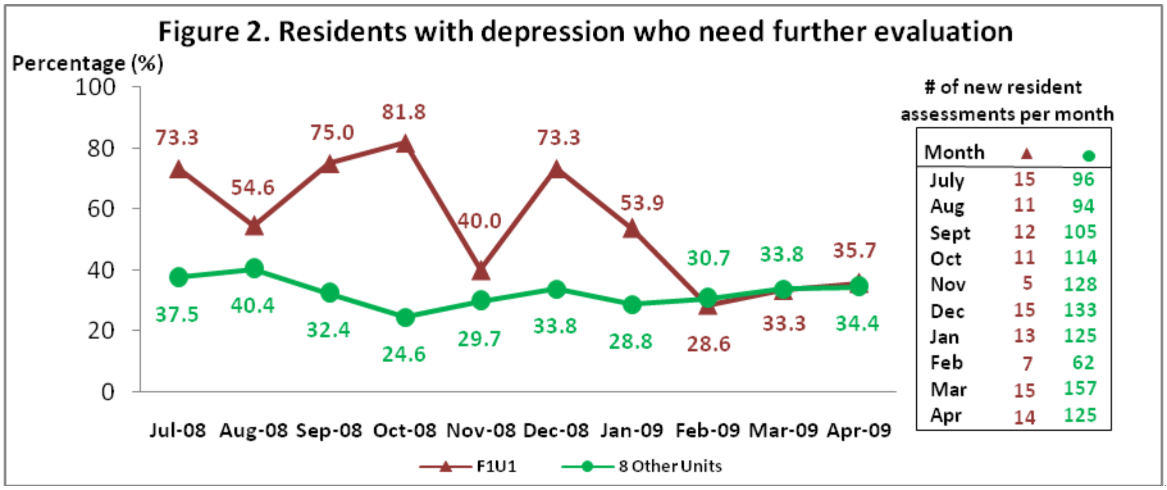

Figure 2. Residents with depression who need further evaluation

- The information in Figure 2 is from the Mood and Behaviour Patterns section (Section E) of the RAI-MDS 2.0, items E1a, E1d, E1f, E1h, E1i, E1l and E1m
- The Depression Rating Scale uses these seven items to provide an overall picture of how much depression the residents have been feeling
- In April, there was almost no change in the proportion of residents in F1U1 who needed to be further evaluated for depression; it was about the same as the other 8 units

➢ FALLS RISK

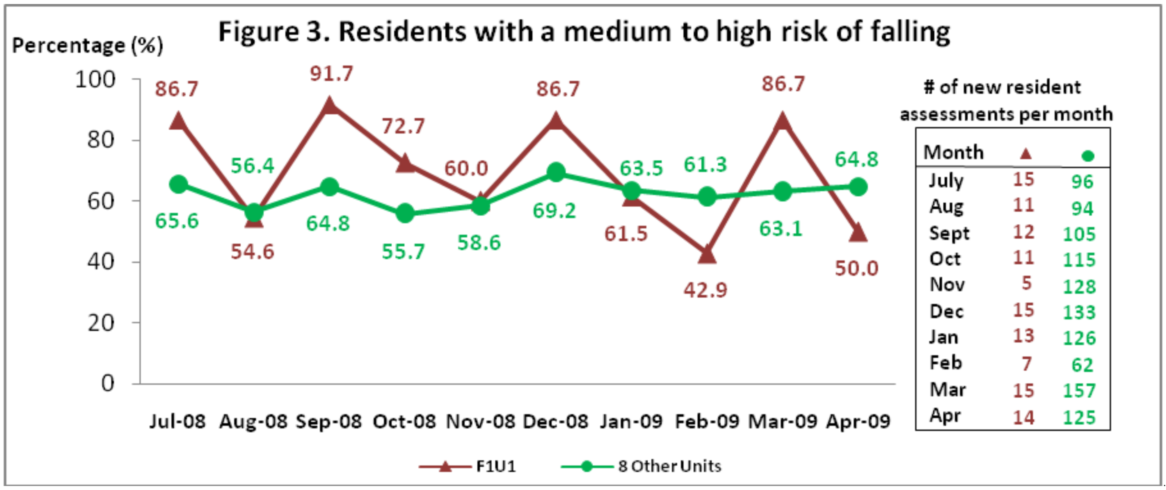

Figure 3. Residents with a medium to high risk of falling

- The information in Figure 3 is from the Physical Functioning and Structural Problems section (Section G) of the RAI-MDS 2.0, items G1ea and G3a and from the Health Conditions section (Section J), items J1f and J1n
- The falls risk scale uses these four items to measure the residents' risk of falling
- In April, there was a large decrease in the proportion of residents in F1U1 who were at a medium to high risk of falling; it was lower than the other 8 units

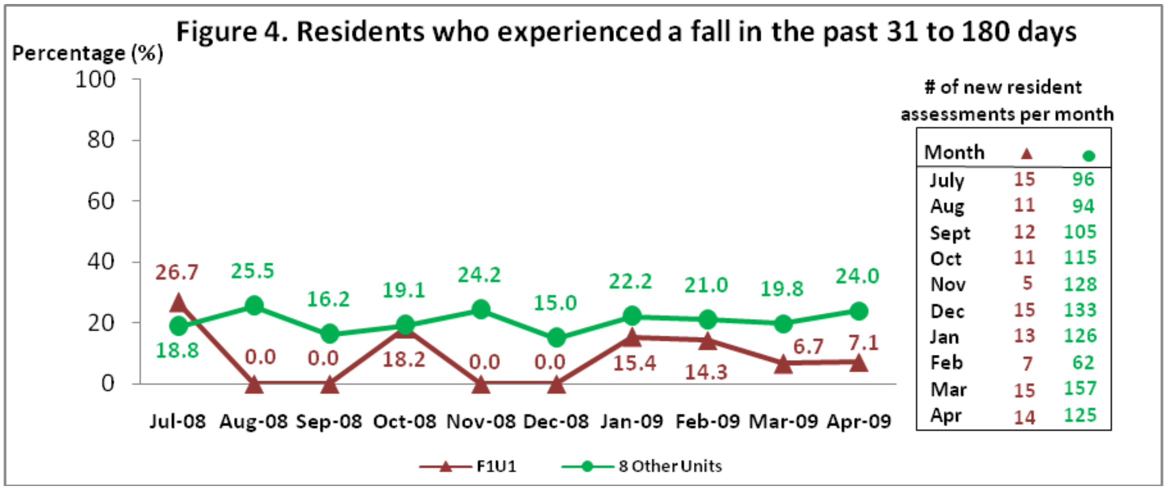

Figure 4. Residents who experienced a fall in the past 31 to 180 days

- The information in Figure 4 is from the Health Conditions section (Section J) of the RAI-MDS 2.0, item J4b
- In April, there was almost no change in the proportion of residents in F1U1 who experienced a fall in the past 31 to 180 days; it was lower than the other 8 units
