# Supplementary material for: Data for improvement and clinical excellence: protocol for an audit with feedback intervention in long-term care
Source: Implement Sci. 2010 Oct 13;5:74. doi: 10.1186/1748-5908-5-74 (PMC2964554; doi:10.1186/1748-5908-5-74)
Supplement: Additional file 3 — Observational checklist. This file contains the checklist used to assess staff behavioural response to the feedback report at the time of distribution. [file 1748-5908-5-74-S3.PDF]

	Month 01				Month 02			
	20-Jan-09	20-Jan-09	22-Jan-09	22-Jan-09	17-Feb-09	17-Feb-09	19-Feb-09	19-Feb-09
	10:30am-12	630pm-8pm	1030am-12	630pm-8pm	10:30am-12	630pm-8pm	1030am-12	630pm-8pm
Staff Member Reading Report (and asking questions)								
Staff Member Reading Report (no questions)								
Staff member putting report in pocket/somewhere else without reading								
Staff member throwing report away without reading								
Staff member throwing report away after reading								
One staff member discussing report with another staff member								
Staff member reads and offers to put it in a team book/common area for others to see								
Report not given directly to an individual (handout person left in a common area, under a door, etc.)								

Other (*please specify)								
No Observation Recorded								
**Total**								
Report not given because individual already seen it (copy in team room or from another staff member)								
*Other:								
Notes								
